# Mr. Atkinson, in Reply

**Published:** 1802-04

**Authors:** James Atkinson


### *To the Editors of the Medical and Physical Journal.*

Gentlemen,

We muſt ever be amuſed with the ſeducing allurements of theory; and every practitioner embraces his own favorite hypotheſis with all the glowing warmth of enthuſiaſtic devotion. If the works of authors had been leſs exuberant in fanciful ideas, there certainly would have followed more practical advantages; but the labour of ſtudy is agreeably relieved, when an enlightened mind enjoys an expanſive and philoſophic view of the various and intereſting phænomena of Nature. The illuſtrious names of Cullen and Darwin will be long remembered, and their works read with delight by ſucceeding generations.

The Controverſy on Apoplexy is yet undecided. The doctrines of Dr. Langſlow I have anſwered.* His defence againſt Mr. Crowfoot appeared to me, with ſome exceptions, to be pregnant with utility and juſt diſcrimination: But as he ſeems diſſatisfied with my opinions, and as the conteſted points between us do not influence any material variation in the mode of treatment of the diſeaſe in queſtion, I ſhall be brief in the ſubſequent obſervations.

I ſtill contend, that "There often occurs Apoplexy without an effuſion, or exudation of *blood*, *or* SERUM, upon the brain; and that when there is *conſiderable* extravaſation, the caſe is commonly fatal." I now ſpeak in general terms of the diſeaſe, without adverting particularly to the caſe related by Dr. Langſlow. Notwithſtanding the abſorbents of the brain have not yet been diſcovered, their exiſtence, from analogy, is generally believed; conſequently they muſt be very minute, and in proportion to their magnitude muſt be the abſorbing power. The abſorbents in other parts of the ſyſtem are comparatively large, and thus admit of a greater quantity of any effuſion or exudation being abſorbed in a given time. The blood, when extravaſated, undergoes a chemical change before it is ſufficiently fluid to be taken up by the lymphatic abſorbents (*Zoonom. vol.* 3), and this muſt *conſiderably impede* the recovery of the patient. The *immediate* alleviation of all the formidable ſymptoms of Apoplexy, is eaſily explained on the principle of a turgeſcency of the arterial or vaſcular ſyſtem of the brain; and this local plethora is uniformly remedied by bliſters, cathartics, and venæſection. In fatal caſes of extravaſation, I conceive that the mouths of the abſorbing veſſels have been choak-

* Medical Journal, Vol. vii. p. 151.

ed up with coagulated blood, which acting as an extraneous body, produces inflammation, and ultimately, death. The causes of death, and of disease, are never allowed to be the same, and I accord with the sentiments of the ingenious Dr. Mossman, that " A sudden effusion of any *large quantity* of fluid upon the brain, is incompatible with animal life." Dr. Langslow does not coincide with Dr. Cullen, that expansion, congestion, or a fulness of the vessels in the head, will produce *compleat* Apoplexy, which I consider to be the most common cause of the disease, even in its most tremendous form. I recollect, when in Edinburgh, seeing a beautiful young lady fall down in an apoplectic fit in the street. Her breathing was loud and stertorous; her eyes were raised and glassy, but stedfastly fixed; and her frame was once or twice for a moment agitated.* It is probable that she might have recovered without any medical aid, as the violence of the paroxysms was greatly diminished before the usual means could be employed. It was in the morning when she was attacked by the fit, and in the evening the surgeon informed me, she was perfectly well. Thus I can hardly believe there was, in this instance, extravasation, exudation, or effusion. Lately I have been favoured with a case, by an eminent physician in this neighbourhood, where paralysis preceded the apoplectic seizure, and which clearly evinces that its fatality was caused by extravasation of blood upon the brain.

R. H. a strong healthy-looking man, after being exhausted with exertion, had profuse sweats, and his limbs affected with incessant trembling. At length the paralysis became universal, which continued two hours, and then he fell down suddenly in a fit of Apoplexy. He had every assistance the most skilful of the Faculty could administer, but death, in defiance of all their efforts, soon closed the scene. Leave could not be obtained to open the body, but a great quantity of blood was observed to have burst from his nose, ears, and mouth. Now I believe† palsy does not very often *precede* Apoplexy; but the eloquent and indefatigable Dr. Darwin infers that Apoplexy may be termed an universal palsy, or a permanent sleep (Zoonom. Vol. 4).

On

* Are, frothing at the mouth, spasms of the muscular system, &c. &c. as represented by Mr. Wilkinson, (Vol. vii. p. 143) the diagnostic symptoms of Apoplexy? Are not they decisively characteristic of Epilepsy?

† I have known a severe hemicrania succeeded by a temporary paralysis. Opium was given in a sufficient dose, so as to produce intoxication; soon after the patient had a few hours long-denied sleep, and in the morning the shooting vertiginous pain was gone.

On the extraordinary doctrines of Mr. Crowfoot, a few words may fuffice. Suppofe we, for a moment, allow that the affection of the ftomach is the predifponent caufe of nervous Apoplexy! And that the diftenfion of that vifcus, from a full meal, compreffing the abdominal aorta, occafions vertigo, ftricture, and oppreffion of the head, fo that the patient drops down fenfelefs to the ground! And that during the exacerbations, an emetic is exhibited! What follows? The increafed ftimulation of the emetic will throw the ftomach into more violent action, and the compreffing power on the aorta will be, for a while, more forcibly applied. The veffels of the head being previoufly turgid and diftended with accumulated blood; what will be the confequence of the additional impetus from this operation? It is true, the fenforial power is much diminifhed after the vomiting has ceafed, but I regard it as a moft objectionable and very injudicious remedy. If Mr. Crowfoot contends that there is no *preffure upon the brain*, I cannot imagine that the cafe is either nervous, fanguineous, or ferous *Apoplexy:* For, certainly, no cafe of *real* Apoplexy *ever occurred* but was caufed by an affection of the brain.

The theories of the moft fatal maladies have been extremely multifarious. In the infancy of medicine, books were crowded with conjectures. The ancient profeffors, however, were uncommonly induftrious. Diffections of the human body were then rarely performed; and although they might not be all men of well cultivated, luxuriant genius, they poffeffed, with their mechanical notions of organized matter, much fagacity and penetration. In a voluminous work, entitled, A Defcription of the Body of Man, publifhed in 1631, I obferve the following *very eccentric* Remarks on Apoplexy, with which I beg to conclude this Communication.

"I know well that when the jugular veins and the carotidall arteries are obftructed, the *caros* Apoplexy and Lethargy doe follow; whence the carotidall artery is called lethargicall and apoplecticall; and Hippocrates ufeth to call that kind of Apoplexy, *intuception of the veines!* Epilepfy, and fuch like difeafes, doe proceed by the obftructing or cimmering of the fpirits, which happeneth when the beginning of the fpinal marrow is choaked by an unnatural confluence of humours unto it. And this opinion is confirmed by the diffection of heads of fuch men as die apoplecticall, in the ventricles of whofe brains there is not found any greater quantity of excrement than is commonly found in other men. Moreover, the caufes of an apoplexy, epilepfy, or falling ficknefs, and the incubus or night mare, are by all phyfitians acknowledged to be, when as phlegme, or craffe and thicke wind, is retained in the ventricles, which ftopping them up either wholly or for the moft part, doe *ftrangle the*

*spirits*

*spirits* therein contained. And I reade in Hippocrates, as in the text of his *Booke de Glandulis*, that the Apoplexy is occafioned by *the corrosion of the braine*." I am, &c.

*March* 9, 1802. JAMES ATKINSON.

